# Identification of differentially expressed genes using an annealing control primer system in stage III serous ovarian carcinoma

**DOI:** 10.1186/1471-2407-10-576

**Published:** 2010-10-22

**Authors:** Yun-Sook Kim, Jin Hwan Do, Sumi Bae, Dong-Han Bae, Woong Shick Ahn

**Affiliations:** 1 Department of Obstetrics and Gynecology, Soonchunhyang University Chunan Hospital, 23-20 Bongmyeong-dong, dongnam-gu, Cheonan-si, Chungcheongnam-do, 330-721, Korea; 2 Cancer Research Institute of Medical Science, The Catholic University of Korea, 505 Banpodong, Seocho-ku, Seoul, 137-040, Korea; 3 Department of Obstetrics and Gynecology, The Catholic University of Korea, 505 Banpodong, Seocho-ku, Seoul, 137-040, Korea

## Abstract

**Background:**

Most patients with ovarian cancer are diagnosed with advanced stage disease (*i.e*., stage III-IV), which is associated with a poor prognosis. Differentially expressed genes (DEGs) in stage III serous ovarian carcinoma compared to normal tissue were screened by a new differential display method, the annealing control primer (ACP) system. The potential targets for markers that could be used for diagnosis and prognosis, for stage III serous ovarian cancer, were found by cluster and survival analysis.

**Methods:**

The ACP-based reverse transcriptase polymerase chain reaction (RT PCR) technique was used to identify DEGs in patients with stage III serous ovarian carcinoma. The DEGs identified by the ACP system were confirmed by quantitative real-time PCR. Cluster analysis was performed on the basis of the expression profile produced by quantitative real-time PCR and survival analysis was carried out by the Kaplan-Meier method and Cox proportional hazards multivariate model; the results of gene expression were compared between chemo-resistant and chemo-sensitive groups.

**Results:**

A total of 114 DEGs were identified by the ACP-based RT PCR technique among patients with stage III serous ovarian carcinoma. The DEGs associated with an apoptosis inhibitory process tended to be up-regulated clones while the DEGs associated with immune response tended to be down-regulated clones. Cluster analysis of the gene expression profile obtained by quantitative real-time PCR revealed two contrasting groups of DEGs. That is, a group of genes including: *SSBP1*, *IFI6 DDT*, *IFI27*, *C11orf92*, *NFKBIA*, *TNXB*, *NEAT1 *and *TFG *were up-regulated while another group of genes consisting of: *LAMB2*, *XRCC6*, *MEF2C*, *RBM5*, *FOXP1*, *NUDCP2*, *LGALS3*, *TMEM185A*, and *C1S *were down-regulated in most patients. Survival analysis revealed that the up-regulated genes such as *DDAH2, RNase K and TCEAL2 *might be associated with a poor prognosis. Furthermore, the prognosis of patients with chemo-resistance was predicted to be very poor when genes such as *RNase K, FOXP1*, *LAMB2 *and *MRVI1 *were up-regulated.

**Conclusion:**

The DEGs in patients with stage III serous ovarian cancer were successfully and reliably identified by the ACP-based RT PCR technique. The DEGs identified in this study might help predict the prognosis of patients with stage III serous ovarian cancer as well as suggest targets for the development of new treatment regimens.

## Background

Ovarian cancer is a complex disease, characterized by successive accumulation of multiple molecular alterations in both the cells undergoing neoplastic transformation and host cells [1]. These anomalies disturb the expression of genes that control critical cell processes, leading to the initiation of tumorigenesis and development. At the time of diagnosis most patients with ovarian cancer have advanced stage disease (*i.e*., stage III-IV) where surgery and chemotherapy results in an approximately 25% overall 5-year survival rate. Consequently, ovarian cancer is the leading cause of death from a gynecological malignancy. Epithelial ovarian cancer (EOC) accounts for 90% of all ovarian cancers; there is significant heterogeneity within the EOC group. For example, histologically defined subtypes such as serous, endometrioid, mucinous, and low- and high-grade malignancies all have variable clinical manifestations and underlying molecular signatures [2].

Gene expression has been extensively applied to screening for the prognostic factors associated with ovarian cancer; identification of such factors would help to determine patient prognosis. Studies have focused on differential gene expression between tumor and normal tissues [3], distinguishing between histological subtypes [4] and identifying differences between invasive tumors and those with low malignant potential [5]. However, to date, the use of differentially expressed genes (DEG) have not been implemented in ovarian cancer therapies; this is mainly because their reliability and validity have not yet been well established. Microarray technology permits large scale analysis of expression surveys to identify the genes that have altered expression as a result of disease. However, microarray data is notorious for its unreliable reproducibility of DEGs across platforms and laboratories, as well as validation problems associated with prognostic signatures [6]. In addition, identification of a gene responsible for a specialized function during a certain biological stage can be difficult to determine because the gene might be expressed at low levels, whereas the bulk of mRNA transcripts within a cell are abundant [7].

To screen DEGs in low concentrations, while minimizing false positive results, the polymerase chain reaction (PCR) based technique has been used. One screening method, differential display, requires PCR using short arbitrary primers. This method is simple, rapid and only requires small amounts of total RNA. However, many investigators have reported significantly high false-positive rates [8] and poor reproducibility of the results [9] because of nonspecific annealing by the short arbitrary primers. Recently, the annealing control primer (ACP) system has been developed; this technique provides a primer with annealing specificity to the template and allows only genuine products to be amplified [10]. The structure of the ACP includes (i) a 3" end region with a target core nucleotide sequence that substantially complements the template nucleic acid of hybridization; (ii) a 5" end region with a non-target universal nucleotide sequence; and (iii) a polydeoxyinosine [poly(dI)] linker bridging the 3" and 5" end sequences. Because of the high annealing specificity during PCR using the ACP system, the application of the ACP to DEG identification generates reproducible, accurate, and long (100 bp to 2 kb) PCR products that are detectable on agarose gels.

In this study, the ACP-based PCR method was used to identify the DEGs of patients with stage III serous ovarian cancer, the findings were compared to normal ovarian tissue. A total of 60 arbitrary ACPs were used and 114 DEGs were identified by sequencing differentially expressed bands. For the confirmation of differential expression of the DEGs, quantitative real-time PCR was performed on 38 selected DEGs; the results showed good agreement with the ACP findings. These results could be used as preliminary data for further study of the molecular mechanism underlying stage III serous ovarian cancer.

## Methods

### Patient information

After obtaining written informed consent from all patients included in the study, samples of primary epithelial ovarian cancer were snap frozen in liquid nitrogen and stored at -80°C. Analysis of tissues from patients was approved by the Institutional Review Board of The Catholic University of Korea (Seoul, Korea). The histopathological diagnoses were determined using the WHO criteria, and the tumor histotype was serous adenocarcinoma in all patients. Classification of cancer stage and grade was performed according to the International Federation of Gynecology and Obstetrics (FIGO). A total of 16 patients with serous ovarian carcinoma were enrolled in this study, all patients were diagnosed as stage IIIC with high-grade cancer (grade 3).

### ACP-based GeneFishing™ reverse transcription polymerase chain reaction

Total RNAs from the ovarian tissues of the serous carcinoma were isolated by gentle homogenization using Trizol^®^. The normal human ovary total RNA was purchased from Stratagene (Total RNA Human Ovary, #540071). The RNA was used for the synthesis of first-strand cDNAs by reverse transcriptase. Reverse transcription was performed 1.5 hours at 42°C in a final reaction volume of 20 μl containing 3 μg of the purified total RNA, 4 μl of 5" reaction buffer (Promega, Madison, WI, USA), 5 μl of dNTPs (2 mmol each), 2 μl of 10 μM dT-ACP1 (5"-CTGTGAATGCTGCGACTACGATIIIII(T)_18_)-3", where "I" represents deoxyinosine), 0.5 μL of RNasin^® ^RNase Inhibitor (40 U/μl, Promega), and 1 μl of Moloney murine leukemia virus reverse transcriptase (200 U/μl, Promega). First-strand cDNAs were diluted by the addition of 80 μL of RNase-free water for the GeneFishing PCR and stored -20°C until use.

**Figure 1:**
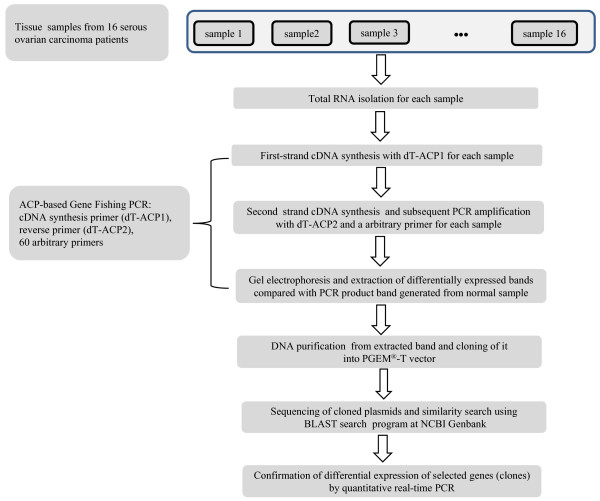
**A schematic diagram of the experimental procedure**.

DEGs were screened by ACP-based PCR method using the GeneFishing™ DEG kits (Seegene, Seoul, South Korea). Briefly, second-strand cDNA synthesis was conducted at 50°C (low stringency) during one cycle of first-stage PCR in a final reaction volume of 49.5 μl containing 3-5 μl (about 50 ng) of diluted first-strand DNA cDNA, 5 μl of 10x PCR buffer plus Mg (Roche Applied Science, Mannheim, Germany), 5 μl of dNTP (each 2 mM), 1 μl of 10 μM dT-ACP2 (5'-CTGTGAATGCTGCGACTACGATIIIII(T)_15_-3"), and 1 μl of 10 μM arbitrary ACP. The tube containing the reaction mixture was kept at 94°C while 0.5 μl of Taq DNA polymerase was added to the reaction mixture (5 U/μl, Roche Applied Science). Sixty PCR reactions for each sample were carried out with 60 arbitrary ACPs, respectively. The PCR protocol for second-strand synthesis was one cycle at 94°C for 1 min, followed by 50°C for 3 min, and 72°C for 1 min. After completion of second-strand DNA synthesis, 40 cycles were performed. Each cycle involved denaturation at 94°C for 40 sec, annealing at 65°C for 40 sec, extension at 72°C for 40 sec, and a final extension at 72°C to complete the reaction. The amplified PCR products were separated in 2% agarose gel and stained with ethidium bromide. The overall scheme of the experiment is shown in Figure 1.

### Cloning and sequencing

The differentially expressed bands were extracted from the gel using the GENCLEAN^® ^II Kit (Q-BIO gene, Carlsbad, CA.,USA), and directly cloned into a TOPO TA^® ^cloning vector (Invitrogen, Karlsruhe, Germany) according to the manufacturer's instructions. The cloned plasmids were sequenced with an ABI PRISM^® ^3100 Genetic Analyzer (Applied Biosystems, Foster City, CA., USA). Complete sequences were analyzed by searching for similarities using the Basic Local Alignment Search Tool (BLAST) search program at the Genbank database of the National Center for Biotechnology Information (NCBI).

### Quantitative real-time polymerase chain reaction and statistical analysis

For the confirmation of the differential expression of DEGs, quantitative real-time PCR was carried out for 38 DEGs selected from 114 DEGs. The concentrations of the reagents were adjusted to reach a final volume of 20 μL containing 5 ng of cDNA template, 10 μl of SYBR^® ^Premis Ex Taq™ II (Takara Bio,Otsu, Japan), 0.4 μl of ROX™ reference Dye II, 0.4 μl of 10 μM forward and reverse primers of DEGs with β-actin as an internal control (Table 1). The cDNA templates were constructed with the total RNA extracted from 16 ovarian cancer tissues and normal human ovary total RNA. The PCR amplification protocol was 50°C for 2 min and 95°C for 10 min followed by 40 cycles of 95°C for 30 sec, 60°C for 30 sec, and 72°C for 30 sec. The real-time PCR analysis was performed on an Applied Biosystems Prism 7900 Sequence Detection System (Applied Biosystems). Relative quantification with the data obtained was performed according to the user's manual. The fold change for gene expression, between the cancer and normal samples, was calculated by using the threshold cycle (C_T_): fold change = 2^-ΔΔ^^*C*^_*T*_, ΔΔ*C*_*T *_= [(C_T _of gene of interest - C_T _of β-actin)_cancer sample_- (C_T _gene of interest - C_T _of β-actin)_normal sample_)]. The fold change was log_2 _transformed for the cluster and survival analysis. The R packages mclust and survival http://www.r-project.org were used for the cluster and survival analysis, respectively.

**Table 1:** Primer sequences of 38 DEGs and *β*-actin used for the quantitative real-time PCR.

DEG	Forward	Reverse	Amplicon size
NM_003143.1	AAAGATCCCTGAATCGTGTGC	TCGCCACATCTCATTAGTTGC	119
AY871274	GTCCACTGCACAGTTCGAGG	GGCCTCCTCTTTGCTGATTC	279
NM_022873.2	CAGAAGGCGGTATCGCTTTTC	CCTGCATCCTTACCCGCATT	89
NM_001355.3	AGCGCCCACTTCTTTGAGTTT	TCCCTATCTTGCCAATCTGCC	106
BC000523	GTGCCTAAGACAGAAATTCGGG	TGCAAGTCTATGTTTGGGTTCAT	174
NM_005532	GCAGCCTTGTGGCTACTCTG	TAGAACCTCGCAATGACAGCC	112
NM_207429.2	GGAGCTCCTTGGAAGTCAGG	GCCAGCAACAGCACTGAGAT	129
BC012823	CGCTCTCTTTTCTCCCGTTT	TCGCAGCATGCTCAACATTA	236
NM_020529	CTCCGAGACTTTCGAGGAAATAC	GCCATTGTAGTTGGTAGCCTTCA	135
NM_001101654	GTGCAATCGCCATTACTGCT	GAATGCAGGGTGTAAGGGGT	248
NM_001867	AAAGGTCTTGGTGAGGTGCC	ACGGACCACAGAGGTTGTGA	119
BC013003	TCAGCACCTTGGAACCTTTGA	AAGACACTCTCTCGGTAGTCATT	100
NM_000978	TCCTCTGGTGCGAAATTCCG	CGTCCCTTGATCCCCTTCAC	119
NM_032470	TTGTCCAGATAGCGGCAAAC	AGCGAGCTCTGGAAGAGGAG	149
NM_013974.1	GGTCGATGGAGTCCGCAAAG	GGTGAAGAGAACGTCAGTGC	100
XM_002345433	CGAGTTCGTGGACCTGTACG	GCCATTAAACCTGCCTGTGA	127
NM_001034996	CATGCCGGAAAATTGGTCGC	CACTGTGCGGAAACTTGAGGA	145
NM_001037637	CATCTCTGGCAGCGAACACTT	AGTCAGACTATCCGCACCAAG	107
BC107854	AGGGGTAAGCTCATCGCAGT	CCGGAAAGTGTCTTCGATCTCA	150
NM_001020	TCGGACGCAAGAAGACAGC	AGCAGCTTGTACTGTAGCGTG	118
NR_003225	GAAACCCAAACCCTCAAGGA	GCACTTGGCTGTCCAGAAGA	247
NM_001004333.3	ATTCCTTGCGCTTATTGAGCC	GCCCCCAGAACATATACAACCT	123
NM_080390	TGCAGGGAGGATCAAAGACA	GGCTCTCCCTCACTCTCTGG	103
NM_013318	AAGCCCTCTGGATCAGCAGT	TCAGTAGGGAGAGGCGAGGT	227
EF177379	GTTTCCAGGCCTTGCTCA	ATTCATGGGCTCTGGAACAA	158
AK026649	GGTTCTCGCTCTTGTCGTGTC	ATATCCTTCGCGTACTGACGG	101
AK302766	TGGCTTTGTAACAAGTGCTGC	CGGAGCTATGTTCCGAAGAATG	168
NM_032682	TCCCGTGTCAGTGGCTATGAT	CTCTTTAGGCTGTTTTCCAGCAT	226
NG_001229	TCATGAGGCCCAGATCAAGA	ACCACGTCCTTCCCTTTCAG	219
AK025219	AGGACCAGAACTGCAAGCTG	GCGCTCTTCCAAGTCAGTGA	155
BC001120	GCAGACAATTTTTCGCTCCA	GCACTTGGCTGTCCAGAAGA	287
NM_032508.1	ATGAACCTGAGGGGCCTCTT	TGATGCCATCCAAACGAAGGG	106
NM_002292.3	ATGCTGGTGGAACGCTCAG	CTCGCCTTCAGTGGATGGC	171
AK293439	GGTCCAGAAGGCTCTCAAGC	GGGCCTCAGGTAATGGTGTT	265
NM_001131005	AGACATCGTGGAGGCATTGA	GTGGCAATAGGTTGGGGTTT	263
NM_201442	CAAGTCCCATACAACAAACTCCA	CAGGAGCAGAAGTAACCACCA	176
BC023599	TCCCTTGTCCGGAGGATATT	TAATGGATTCAATCATCTTTATTAACC	164
NM_001100167	TCTGAAGAGTCCCCCAAATG	AATCCAGCACTTCCTCTCCA	209
*β*-actin	GGCTGTATTCCCCTCCATCG	CCAGTTGGTAACAATGCCATGT	154

## Results

### Differentially expressed genes (DEGs) in stage III serous ovarian cancers

**Figure 2:**
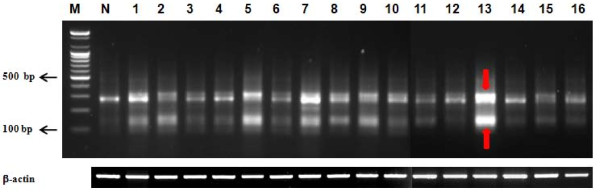
**An example of GeneFishing™ using an arbitrary ACP in combination with an oligo (dT) ACP as indicated in the Methods section**. M and N represents a 100-bp size marker generated by Forever 100-bp Ladder Personalizer (Seegene, Seoul, South Korea) and a normal sample, respectively. The lanes 1-16 include each of the cancer samples from the 16 patients. The bands showing a clear difference between the normal and cancer samples, here marked with a red arrow, were excised from the gel for further cloning and sequencing.

The patients included in this study ranged in age between 38 and 69 (mean age 53.3 ± 7.5 years). All 16 patients had a diagnosis of FIGO stage III papillary serous ovarian carcinoma. To identify genes that showed a predominant change of expression in patients with stage III serous ovarian cancer, the total RNAs from 16 serous ovarian tissues of stage III and the normal human ovary total RNA (Stratagene, #540071) were individually subjected to ACP-based RT PCR analysis using a combination of 60 arbitrary primers and two anchored oligo (dT) primers (dT-ACP1 and dT-ACP2). All PCR amplicons were compared on agarose gels (Figure 2). When the bands generated by the normal sample showed a clear difference compared to the bands generated on the cancer sample, the band was defined as a differentially expressed band. After all poor appearing bands were excluded, the differentially expressed bands were extracted, amplified using the TOPO TA Cloning Kit (Invitrogen, Cat. #K4500-1) and sequenced. The sequences of 114 DEGs were obtained. The DNA sequence of each DEG was analyzed by searching for similarities using the BLASTX program at the Genbank database (NIH, MD, USA). Table 2 shows the 114 DEGs assessed by Genbank and the best homologues.

**Table 2:** Annotation of the 114 DEGs by the BLAST search.

Clone name	Annotation	**GenBank accession no**.	E-value
U1	Homo sapiens acidic (leucine-rich) nuclear phosphoprotein 32 family, member B	BC013003	4.00E-112
U2	Homo sapiens adaptor-related protein complex 3, delta 1 subunit (AP3D1), transcript variant 2	NM_003938	6.00E-10
U3	Homo sapiens adenine phosphoribosyltransferase (APRT), transcript variant 2	NM_001030018	7.00E-08
U4	Homo sapiens genomic DNA, chromosome 11q, clone:CMB9-1B14, complete sequences	AP000659	4.00E-73
U5	Homo sapiens chromosome 19 open reading frame 53 (C19orf53)	NM_014047	1.00E-19
U6	Homo sapiens chromosome 1 open reading frame 115 (C1orf115)	NM_024709	5.00E-24
U7	Homo sapiens coatomer protein complex, subunit alpha (COPA), transcript variant 1	NM_001098398	4.00E-22
U8	Homo sapiens dimethylarginine dimethylaminohydrolase 2 (DDAH2)	NM_013974.1	2.00E-123
U9	Homo sapiens dimethylarginine dimethylaminohydrolase 2	BC001435	9.00E-168
U10	Homo sapiens dynein, light chain, LC8-type 1 (DYNLL1), transcript variant 3	NM_003746	0.00E+00
U11	Homo sapiens ferritin, light polypeptide	BC004245	0.00E+00
U12	Homo sapiens glutamic-oxaloacetic transaminase 2, mitochondrial (aspartate aminotransferase 2) (GOT2), nuclear gene encoding mitochondrial protein	NM_002080	7.00E-21
U13	Homo sapiens sarcoma antigen NY-SAR-48	BC040564	3.00E-95
U14	Homo sapiens heat shock protein 90 kDa alpha (cytosolic), class B member 1 (HSP90AB1)	NM_007355.2	0.00E+00
U15	Homo sapiens interferon, alpha-inducible protein 27 (IFI27), transcript variant 2	NM_005532	6.00E-99
U16	Homo sapiens interferon, alpha-inducible protein 6 (IFI6), transcript variant 3	NM_022873.2	2.00E-35
U17	Homo sapiens iron-responsive element binding protein 2 (IREB2)	NM_004136	5.00E-66
U18	Homo sapiens keratinocyte associated protein 2	BC029806	5.00E-71
U19	PREDICTED: Homo sapiens similar to ribosomal protein S21, transcript variant 2 (LOC100291837)	XM_002345433	1.00E-100
U20	Homo sapiens mesothelin (MSLN), transcript variant 1	NM_005823	0.00E+00
U21	Homo sapiens nuclear factor of kappa light polypeptide gene enhancer in B-cells inhibitor, alpha (NFKBIA)	NM_020529	1.00E-95
U22	Homo sapiens nuclear distribution gene C homolog (A. nidulans) pseudogene 2 (NUDCP2) on chromosome 2	NG_001229	1.00E-101
U23	Homo sapiens PRP8 pre-mRNA processing factor 8 homolog (S. cerevisiae) (PRPF8)	NM_006445.3	4.00E-110
U24	Homo sapiens RAB5B, member RAS oncogene family (RAB5B)	NM_002868	2.00E-123
U25	Homo sapiens cDNA FLJ76524 complete cds	AK289930	2.00E-79
U26	Homo sapiens cell growth-inhibiting protein 34 mRNA, complete cds	AY871274	0.00E+00
U27	Homo sapiens ribosomal protein L23 (RPL23)	NM_000978	1.00E-87
U28	Homo sapiens ribosomal protein L6 pseudogene 27 (RPL6P27) on chromosome 18	NG_009652	2.00E-111
U29	Homo sapiens ribosomal protein S8 (RPS8)	NM_001012	2.00E-67
U30	Homo sapiens TRK-fused gene	BC023599	4.00E-79
U31	Homo sapiens ribosomal protein S8	BC070875	1.00E-60
U32	Homo sapiens mRNA similar to eukaryotic translation initiation factor 3, subunit 7 (zeta, 66/67 kD)	BC011740	8.00E-142
U33	Homo sapiens ribosomal protein S24	BC000523	4.00E-45
U34	Homo sapiens cDNA, FLJ18539	AK311497	4.00E-26
U35	Homo sapiens cDNA clone IMAGE:2822193	BC005845	8.00E-155
U36	Homo sapiens ATPase, H+ transporting, lysosomal 14 kDa, V1 subunit F	BC107854	3.00E-147
U37	Homo sapiens cytochrome c oxidase subunit VIIc (COX7C), nuclear gene encoding mitochondrial protein	NM_001867	5.00E-145
U38	Homo sapiens tenascin XB (TNXB), transcript variant XB-S	NM_032470	0.00E+00
U39	Homo sapiens septin 9 (SEPT9) on chromosome 17	NG_011683	5.00E-45
U40	Homo sapiens chromosome 11 open reading frame 92 (C11orf92)	NM_207429.2	3.00E-45
U41	Homo sapiens D-dopachrome tautomerase (DDT), transcript variant 1	NM_001355.3	5.00E-94
U42	Homo sapiens single-stranded DNA binding protein 1 (SSBP1)	NM_003143.1	0.00E+00
D1	Homo sapiens actin, beta (ACTB),	NM_001101.2	0.00E+00
D2	Homo sapiens acidic (leucine-rich) nuclear phosphoprotein 32 family, member B (ANP32B)	NM_006401.2	4.00E-115
D3	Homo sapiens Rho guanine nucleotide exchange factor (GEF) 17 (ARHGEF17)	NM_014786	7.00E-37
D4	Homo sapiens ATPase, H+ transporting, lysosomal 13kDa, V1 subunit G1	BC003564	0.00E+00
D5	Homo sapiens UDP-Gal:betaGal beta 1,3-galactosyltransferase polypeptide 6 (B3GALT6)	NM_080605	8.00E-29
D6	Homo sapiens HLA-B associated transcript 2-like 1 (BAT2L1)	NM_013318	4.00E-121
D7	Homo sapiens cDNA FLJ77629 complete cds, highly similar to Homo sapiens bone marrow stromal cell antigen 2 (BST2)	AK291099	0.00E+00
D8	Homo sapiens basic transcription factor 3 (BTF3), transcript variant 1	NM_001037637	0.00E+00
D9	Homo sapiens complement component 1, s subcomponent (C1S), transcript variant 1	NM_201442	0.00E+00
D10	Homo sapiens CD74 molecule, major histocompatibility complex, class II invariant chain (CD74), transcript variant 1	NM_001025159.1	8.00E-179
D11	Homo sapiens chloride intracellular channel 1	BC064527	2.00E-143
D12	Homo sapiens CXXC finger 1 (PHD domain) (CXXC1), transcript variant 1	NM_001101654	1.00E-180
D13	Homo sapiens early growth response 1 (EGR1)	NM_001964.2	0.00E+00
D14	Homo sapiens Finkel-Biskis-Reilly murine sarcoma virus (FBR-MuSV) ubiquitously expressed (FAU)	NM_001997	5.00E-99
D15	Homo sapiens F-box protein Fbx7 (FBX7) mRNA, complete cds	AF129537	2.00E-105
D16	Homo sapiens Fc fragment of IgG, receptor, transporter, alpha	BC008734	1.00E-79
D17	Homo sapiens forkhead box P1 (FOXP1), transcript variant 1	NM_032682	2.00E-130
D18	Homo sapiens guanine nucleotide binding protein (G protein), beta polypeptide 1	BC004186	1.00E-55
D19	Homo sapiens H19, imprinted maternally expressed transcript (non-protein coding) (H19), non-coding RNA	NR_002196	2.00E-34
D20	Homo sapiens high density lipoprotein binding protein (HDLBP), transcript variant 2	NM_203346.2	0.00E+00
D21	Homo sapiens cDNA FLJ52975 complete cds, highly similar to Heterogeneous nuclear ribonucleoproteins C	AK299923	0.00E+00
D22	Homo sapiens cDNA clone IMAGE:3898245	BC010864	1.00E-138
D23	Homo sapiens cDNA fis, A-KAT03057, highly similar to Homo sapiens mitochondrion, ATP synthase 6	AK026530	0.00E+00
D24	Homo sapiens cDNA: FLJ21566 fis, clone COL06467	AK025219	0.00E+00
D25	Homo sapiens cDNA: FLJ22996 fis, clone KAT11938	AK026649	4.00E-132
D26	Homo sapiens coiled-coil-helix-coiled-coil-helix domain containing 1 (CHCHD1)	NM_203298.1	0.00E+00
D27	Homo sapiens mRNA similar to guanine nucleotide binding protein-like 1	BC048213	6.00E-29
D28	Homo sapiens mRNA similar to ribosomal protein L30	BC012823	2.00E-131
D29	Homo sapiens ribosomal protein, large, P1	BC053844	5.00E-152
D30	Homo sapiens sarcoma antigen NY-SAR-71 mRNA, partial cds	AY211920	1.00E-138
D31	Homo sapiens tumor necrosis factor, alpha-induced protein 1 (endothelial)	BC006208	8.00E-116
D32	Homo sapiens zinc finger, FYVE domain containing 20	BC021246	1.00E-138
D33	Homo sapiens heat shock factor binding protein 1 (HSBP1)	NM_001537	1.00E-121
D34	Homo sapiens HtrA serine peptidase 1 (HTRA1) on chromosome 10	NG_011554	0.00E+00
D35	Human mitochondrial specific single stranded DNA binding protein mRNA, complete cds	M94556	1.00E-146
D36	Homo sapiens immunoglobulin kappa constant	BC095490	0.00E+00
D37	Homo sapiens immunoglobulin lambda locus, mRNA (cDNA clone MGC:88803 IMAGE:4765294), complete cds	BC073786	3.00E-101
D38	Homo sapiens jun B proto-oncogene (JUNB) gene, complete cds	AY751746	2.00E-20
D39	Homo sapiens L antigen family, member 3, mRNA (cDNA clone MGC:23038 IMAGE:4899044), complete cds	BC015744	6.00E-29
D40	Homo sapiens laminin, beta 2 (laminin S) (LAMB2)	NM_002292.3	0.00E+00
D41	Homo sapiens lectin, galactoside-binding, soluble, 1, mRNA (cDNA clone MGC:1818 IMAGE:2967299), complete cds	BC001693	5.00E-52
D42	Homo sapiens lectin, galactoside-binding, soluble, 3, mRNA (cDNA clone MGC:2058 IMAGE:3050135), complete cds	BC001120	0.00E+00
D43	Homo sapiens lectin, galactoside-binding, soluble, 3 (LGALS3), transcript variant 2, non-coding RNA	NR_003225	0.00E+00
D44	Homo sapiens mRNA for LGALS3 protein variant protein	AB209391	0.00E+00
D45	PREDICTED: Homo sapiens similar to ribosomal protein L13a (LOC100293761)	XM_002344734	8.00E-51
D46	Homo sapiens mediator complex subunit 1 (MED1)	NM_004774	1.00E-62
D47	Homo sapiens mediator complex subunit 14 (MED14)	NM_004229	3.00E-49
D48	Homo sapiens myocyte enhancer factor 2C (MEF2C), transcript variant 2	NM_001131005	1.00E-99
D49	Homo sapiens murine retrovirus integration site 1 homolog (MRVI1), transcript variant 4	NM_001100167	0.00E+00
D50	Homo sapiens myosin light chain kinase (MYLK), transcript variant 3A	NM_053027	2.00E-97
D51	Homo sapiens cDNA FLJ53659 complete cds, highly similar to Myosin light chain kinase, smooth muscle	AK300610	0.00E+00
D52	Homo sapiens nuclear receptor co-repressor 2 (NCOR2), transcript variant 1	NM_006312.3	0.00E+00
D53	Homo sapiens nuclear enriched abundant transcript 1 (NEAT1) mRNA, complete sequence	EF177379	1.00E-80
D54	Homo sapiens trophoblast MHC class II suppressor mRNA, complete sequence	AF508303	1.00E-67
D55	Homo sapiens cDNA FLJ53682 complete cds, highly similar to RNA-binding protein 5	AK302766	2.00E-153
D56	Homo sapiens RNA binding motif protein 5	BC002957	2.00E-153
D57	Homo sapiens RNA binding motif protein 8A (RBM8A)	NM_005105.2	1.00E-135
D58	Homo sapiens ribonuclease, RNase K (RNASEK)	NM_001004333.3	6.00E-99
D59	Homo sapiens ribosomal protein L14 (RPL14), transcript variant 1	NM_001034996	0.00E+00
D60	Homo sapiens ribosomal protein L27a (RPL27A)	NM_000990	4.00E-171
D61	Homo sapiens ribosomal protein L29 (RPL29)	NM_000992.2	6.00E-154
D62	Homo sapiens ribosomal protein S16 (RPS16)	NM_001020	7.00E-136
D63	Homo sapiens ribosomal protein S20 (RPS20), transcript variant 2	NM_001023	0.00E+00
D64	Homo sapiens ribosomal protein S21 (RPS21)	NM_001024	8.00E-122
D65	Homo sapiens single-stranded DNA binding protein 1	BC000895	1.00E-146
D66	Homo sapiens transcription elongation factor A (SII)-like 2 (TCEAL2)	NM_080390	0.00E+00
D67	Homo sapiens tubulointerstitial nephritis antigen-like 1 (TINAGL1)	NM_022164	2.00E-153
D68	Homo sapiens transmembrane protein 185A (TMEM185A)	NM_032508.1	0.00E+00
D69	Homo sapiens cDNA FLJ57738 complete cds, highly similar to Translationally-controlled tumor protein	AK296587	2.00E-104
D70	Homo sapiens X-ray repair complementing defective repair in Chinese hamster cells 6 (XRCC6)	NM_001469.3	0.00E+00
D71	Homo sapiens cDNA FLJ53970 complete cds, highly similar to ATP-dependent DNA helicase 2 subunit 1	AK293439	0.00E+00
D72	Homo sapiens phosphodiesterase 7B (PDE7B) on chromosome 6	NG_011994	3.00E-41

The prefix U and D in the clone name represent the clone from the up- and down-regulated bands in the agarose gel. The expression of DEGs marked in bold was further confirmed by quantitative real-time PCR.

The DEGs identified from the clones that were up-regulated included: AP000659 (U4, *ARHGEF12*), NM_013974.1 (U8, *DDAH2*), NM_022873.2 (U16, *IFI6*), NM_020529 (U21, *NFKBIA*), BC023599 (U30, *TFG*), and NG_011683 (U39, *SEPT9*). The up-regulation of *SEPT9 *mRNA was reported in a bank of ovarian tumors, which included benign, borderline and malignant tumors [11]. The genes including *DDAH2*, *IFI6 *and *NFKBIA *are known to be involved in the apoptosis inhibitory process while *ARHGEF12 *and *TGF *have been implicated in signaling pathways. The DEGs identified from down-regulated clones included: AK291099 (D7, *BST2*), NM_001025159.1 (D10, *CD74*), BC004186 (D18, *GNB1*), NG_011554 (D34, *HTRA1*), BC095490 (D36, *IGKC*), and BC001693 (D41, *LGALS1*). The down-regulation of *HTRA1 *was associated with ovarian cancer metastasis [12]. The genes including *BST2, CD74 *and *IGKC *were associated with the immune system; while *GNB1 and LGALS1 *were related to the modulation of cell-cell interaction and the G protein coupled receptor protein signaling pathway, respectively.

### Confirmation of ACP observation by quantitative real-time PCR and cluster analysis

**Figure 3:**
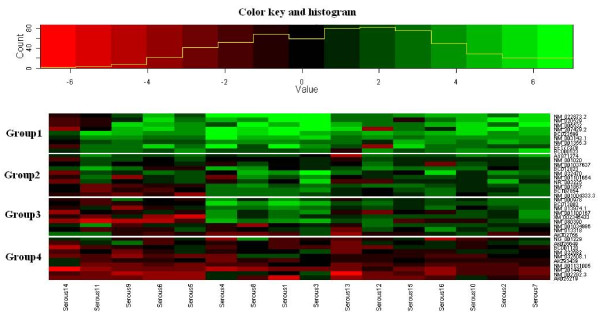
**Clustering of the log_2 _expression ratios of the cancer to normal samples measured by the quantitative real-time PCR for the 38 DEGs from the 16 patients and its representation as a heat map**. Patients are ordered along the *X*-axis and genes along the *Y*-axis.

To confirm the efficacy of the ACP system, confirmation of the differential expression of DEGs was performed with quantitative real-time PCR for 38 DEGs selected from the total 114 DEGs using a specific primer pair for each gene (Table 1 and Additional file 1). The expression ratio of the cancer to normal sample was calculated by using C_T _and then was log_2 _transformed (see Methods section for detail). The DEGs were considered differentially expressed if the log_2 _ratio was > 1.0 or < -1.0. Differential expression was clearly observed in all 38 DEGs, which indicates a high reliability of the ACP system (Figure 3).

For the detection of more conserved expression patterns in patients with stage III serous ovarian cancer, cluster analysis was performed using the R package mclust http://www.r-project.org. Thirty eight DEGs were divided into four groups according to their expression profiles with assignment of each gene to a group (Figure 3). A clear contrast in expression patterns was noted between groups 1 and 4. That is, the overall up- and down-regulation in group 1 was 86.8% and 5.6%, respectively; the overall up- and down-regulation in group 4 was 9.3% and 60.3%, respectively, when up-regulation corresponds to log_2 _ratio > 1 and down-regulation to log_2 _ratio < -1 (Table 3). This regulation pattern in each group was well maintained at various thresholds used for definition of differential expression. These findings suggest that the genes in groups 1 and 4 might be used as potential markers for prognosis in patients with stage III serous ovarian cancer. The group 1 consisted of: NM_003143.1 (*SSBP1*), NM_022873.2 (*IFI6*), NM_001355.3 (*DDT*), NM_005532 (*IFI27*), NM_207429.2 (*C11orf92*), NM_020529 (*NFKBIA*), NM_032470 (*TNXB*), EF177379 (*NEAT1*) and BC02359 (*TFG*); group 4 was composed of: NM_002292.3 (*LAMB2*), AK025219, AK293439 (*XRCC6*), NM_001131005 (*MEF2C*), AK302766 (*RBM5*), NM_032682 (*FOXP1*), NG_001229 (*NUDCP2*), BC001120 (*LGALS3*), NM_032508.1 (*TMEM185A*), and NM_201442 (*C1S*) (Table 3). All genes except for *NEAT1 *in group 1 were identified from up-regulated clones while all genes except for *NUDCP2 *in group 4 were identified from down-regulated clones (Tables 2 and 3). These results were in good agreement with the ACP findings.

**Table 3:** Clustering of 38 DEGs according to the expression profiles of 16 patients with serous ovarian cancer; Up-regulation corresponds to log_2 _ratio > threshold while down-regulation to log_2 _ratio < minus value of threshold.

Threshold	Group 1		Group 2		Group 3		Group 4	
	
	up-regulated	down-regulated	up-regulated	down-regulated	up-regulated	down-regulated	up-regulated	down-regulated
0.5	88.9%	6.9%	68.8%	15.6%	63.9%	22.2%	19.4%	68.8%
1.0	86.8%	5.6%	58.8%	12.5%	55.6%	18.1%	9.3%	60.3%
1.5	84.0%	4.2%	45.6%	7.5%	48.6%	13.2%	6.3%	46.3%
2.0	77.1%	3.5%	31.9%	6.9%	41.0%	11.8%	3.1%	33.1%

Accession no. (gene symbol)	NM_003143.1 (SSBP1)		AY871274 (RPL11)		NM_001867 (COX7C) BC013003 (ANP32B)		AK302766 (RBM5) NM_032682 (FOXP1)	
	NM_022873.2 (IFI6)		BC000523 (RPS24)		BC013003 (ANP32B)		NM_032682 (FOXP1)	
	NM_001355.3 (DDT)		BC012823		NM_000978 (RPL23)		NG_001229 (NUDCP2)	
	NM_005532(IFI27)		NM_001101654(CXXC1)		NM_013974.1 (DDAH2)		AK025219	
	NM_207429.2 (C11orf92)		NM_001034996(RPL14)		XM_002345433		BC001120 (LGALS3) NM_032508.1 (TMEM185A)	
	NM_020529(NFKBIA)		NM_001037637(BTF3)		BC107854 (ATP6V1F)			
	NM_032470(TNXB)		NM_001020(RPS16)		NM_001004333.3 (RNASEK)		NM_002292.3 (LAMB2)	
	EF177379 (NEAT1)		NR_003225(LGALS3)		NM_080390(TCEAL2)		AK293439 (XRCC6)	
	BC02359 (TFG)		NM_013318(BAT2L1) AK026649		NM_001100167(MRVI1)		NM_001131005(MEF2C) NM_201442 (C1S)	

The percentage of up- and down-regulation was calculated for each group.

### Survival analysis

**Figure 4:**
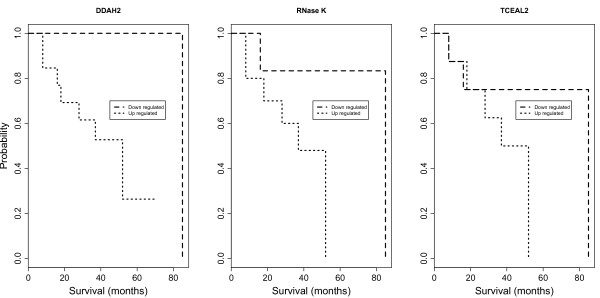
**Kaplan-Meier estimates of overall survival stratified by up- and down-regulation for three genes including *DDAH2*, *RNase K *and *TCEAL2***.

The Kaplan-Meier method was performed using the 38 DEGs that were up- and down-regulated. The up and down regulated genes were considered according to the log_2 _expression ratio > 0 and < 0, respectively. A significant difference, in the overall survival (*p *values < 0.05) between the up- and down-regulated group, was observed for three DEGs including NM_013974.1 (dimethylarginine dimethylaminohydrolase 2, *DDAH2*), NM_001004333.3 (ribonuclease K, *RNase K*) and NM_080390 (transcription elongation factor A (SII)-like 2, *TCEAL2*) (Figure 4). The overall survival decreased with up-regulation of these genes. *DDAH2 *predominates in the vascular endothelium, which is the site of endothelial nitric oxide synthase (eNOS) expression [13,14]. *TCEAL2 *is a nuclear phosphoprotein that modulates transcription in a promoter context-dependent manner and has been recognized as an important nuclear target for intracellular signal transduction. The function of *RNase K *is unknown but might be related to an enhanced degradation of the tumor suppressor gene mRNAs, which leads to the development of cancer. The up-regulation of these three genes was observed in more than 60% of the total number of patients.

**Figure 5:**
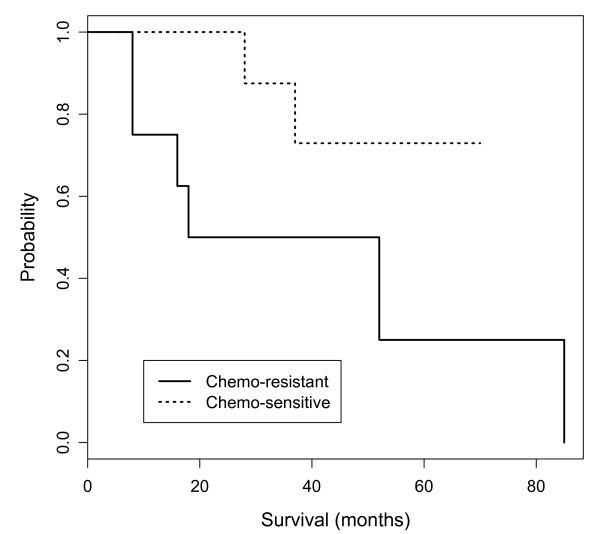
**Kaplan-Meier estimates of overall survival stratified by chemo-resistance**.

**Figure 6:**
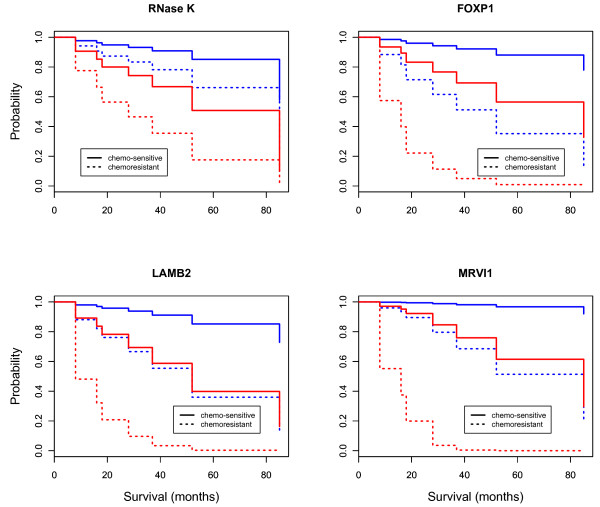
**The overall survival estimated by the Cox proportional hazards multivariate model including gene expression and chemo-resistance**. The up- and down-regulation is represented by red and blue, respectively.

The survival analysis also was performed for chemo-resistance. Following debulking surgery, all patients received platinum-based chemotherapy, considered the standard of care for patients with advanced ovarian cancer. The patients that had either progression during chemotherapy or relapse within six months of treatment were considered chemo-resistant. Among 16 patients, eight were classified as chemo-resistant and the others were categorized as chemo-sensitive. The difference in overall survival between these two groups was significant (*p *value < 0.05, Figure 5). The shorter survival time of patients with chemo-resistance is consistent with a prior report [15]. To consider chemo-resistance and gene expression simultaneously, in the prediction of overall survival, the Cox multivariate analysis was carried out with the expression information of 38 DEGs and chemo-resistance information from 16 patients. Multivariate analysis demonstrated a significant difference in overall survival between the chemo-resistant and sensitive groups for four DEGs including: NM_001004333.3 (*RNase K*), NM_032682 (forkhead box transcription factor family, *FOXP1*), NM_002292.3 (a family of extracellular matrix glycoproteins, LAMB2), and NM_001100167 (murine retrovirus integration site 1 homolog, *MRVI1*) (*p *values < 0.05, Figure 6). The *RNase K *showed significance in both univariate (gene expression) and multivariate (gene expression and chemo-resistance) analysis while *DDAH2 and TCEAL2 *showed significance only in univariate analysis. This was mainly due to the similar proportion of up- and down-regulation of *DDAH2 and TCEAL2 *between the chemo-resistant and chemo-sensitive groups. The overall survival of patients with chemo-resistance was significantly decreased with up-regulation of these four genes; while the chemo-sensitive patients had down-regulation of these genes and a good prognosis. FOXP1 has a diverse repertoire of functions ranging from the regulation of B-cell development and monocyte differentiation to the facilitation of cardiac valve and lung development [16,17]. LAMB2 might be involved in the cell adhesion or motility of prostate cancer cells [18]. The down-regulation of *FOXP1 *and *LAMB2 *was observed in more than 60% of all patients while MRVI was up-regulated among 63%.

## Discussion

Ovarian cancer is the most common cause of death among all gynecological malignancies. The five-year survival rates in patients with ovarian cancer are about 80-90% for stage Ia-Ic, 70-80% for stage IIa-IIc, 30-50% for stages IIIa-IIIc and 13% for stage IV [19]. The high rate of death is due to the fact that most of patients (>60%) present with advanced stage disease (FIGO stages III/IV). Despite an initial response rate of 65%-80% to first-line chemotherapy, most ovarian carcinomas relapse. Acquired resistance to further chemotherapy is generally responsible for treatment failure. Several studies have sought to identify gene expression signatures that correlate with clinical outcome to identify those genes that are associated with survival and relapse and to use as predictive biomarkers for response to chemotherapy [20-22].

There are many types of ovarian cancer. EOC accounts for 85%-90%; half of such cases are serous EOC. As with many cancers thought to be of epithelial origin, it is important to establish an appropriate control for evaluating differential gene expression between "normal" and cancer. Expression profiling studies of ovarian cancer have relied on a variety of sources of normal cells or comparison with tumors, including whole ovary samples (WO), ovarian surface epithelium (OSE), exposed to short-term culture, and immortalized OSE cell lines (IOSE) [23]. Direct comparison of the gene expression profiles generated from OSE brushings, WO samples, short-term cultures of normal OSE (NOSE), and telomerase-immortalized OSE (TIOSE) cell lines revealed that these "normal" samples formed robust, but very distinct groups in hierarchical clustering [24]. These indicate that the selection of normal control to compare epithelial ovarian samples in microarray studies can strongly influence the genes that are identified as differentially expressed. In this study, the total RNA from WO (Stratagene, http://www.stratagene.com) was used as control. WO samples have potential to obscure epithelial pattern due to large amounts of stroma, but they offer the advantages of avoidance of exposure to culture conditions and identification of differential gene expression pattern between tumor and normal tissue [25].

A total of 114 DEGs from patients with serous ovarian cancer stage III were identified using the ACP-based GeneFishing™ PCR system, which uses primers that anneal specifically to the template and allows only genuine products to be amplified. As the GeneFishing™ system is based on PCR, it can overcome the difficulty in identifying the genes responsible for a specialized function during a certain biological stage; this is because the gene is expressed at low levels, whereas most mRNA transcripts within a cell are abundantly expressed. Among the 114 DEGs, 42 were identified as up-regulated clones while 72 were down-regulated clones. These DEGs were involved in a variety of biological processes including apoptosis, signal transduction and the immune response. Apoptosis inhibitory processes were associated with genes such as *NFKBIA*, *DDAH2 *and *IFI6 *identified from up-regulated clones; while the immune system associated genes such as *IGKC*, *CD74 *and *BST2 *were found in down-regulated clones. The differential expression of DEGs identified by the Genefishing™ system showed good agreement with the results of the quantitative real-time PCR.

Cluster analysis based on gene expression profiles identified two groups showing a contrast in the expression pattern. That is, one group including: *SSBP1*, *IFI6 DDT*, *IFI27*, *C11orf92*, *NFKBIA*, *TNXB*, *NEAT1 *and *TFG*, was up-regulated in most patients with stage III serous ovarian cancer (Figure 3 and Table 3). These genes might be utilized as potential targets in patients with stage III serous ovarian cancer. *IFI6 *is involved in apoptosis inhibitory activity while *TGF *is implicated in up regulation of the I-κ B kinase/NF-κ B cascade. *TNXB *encodes TNX, a protein of unknown function that is mainly expressed in the peripheral nervous system and muscles. The promotion of tumor invasion and metastasis has been reported in mice deficient in TNX through the activation of the matrix metalloproteinase 2 (*MMP2*) and *MMP9 *genes [26]. This indicates that the up-regulation of *TNXB*, in patients with advanced stage of ovarian cancer, might induce low expression of *MMP2 *and *MMP9*. The decrease of MMP2 has been reported in liver metastases in advanced colorectal cancers [27]. Another group consists of *LAMB2*, *XRCC6*, *MEF2C*, *RBM5*, *FOXP1*, *NUDCP2*, *LGALS3*, *TMEM185A*, and *C1S*, which was down-regulated in most patients with stage III serous ovarian cancer (Figure 4 and Table 3). The down-regulation of *LAMB2 *and *MEF2C *might be involved with cell adhesion or motility in invasive prostate cancer cells [18] and apoptosis via BCL2 transformation [28], respectively. *FOXP1 *is a potential therapeutic target in cancer and can be considered either an oncogene and/or a tumor suppressor gene [29]. That is, its over-expression confers a poor prognosis in a number of types of lymphomas while the loss of its expression in breast cancer is associated with a poor outcome. The function of *FOXP1 *in serous ovarian cancer remains unclear. *LGALS3 *encoding galectin-3 has been implicated in advanced stage disease [30]. The distribution of galectin has been associated with stage III-V with cellular changes such as dysplasia, cancer cells' nest formation, breakage of the basement membrane, and infiltration of cells into non-native tissue. However, 13 out of the total 16 patients with stage III serous ovarian cancer had down-regulation of *LGALS3*. This is consistent with the report by van den Brule *et al*. [31] that showed that galectin-3 expression was decreased in 67% of cases compared to the normal epithelial cells. However, it conflicts with the observations of Lurisci *et al*. [32] that galectin-3 serum levels in patients with ovarian cancer were significantly elevated. The expression of *LGALS3 *might be affected by the stage of ovarian cancer.

The overall survival estimated by the Kaplan-Meier method was significantly different between the up- and down-regulated patient cohort with regard to three genes including *DDAH2, RNase K *and *TCEAL2*. The up-regulation of these genes was associated with a shorter overall survival (Figure 4). In rapidly growing cells like tumor cells, the activity of RNases is decreased [33], as dictated by the requirement of significant amounts of RNA for protein synthesis. However, high RNase activity has been reported in chronic myeloid leukemia [34] and pancreatic carcinoma [35]. In this study, 70% of the patients with stage III serous ovarian cancer showed up-regulation of *RNase K*. The function of *RNase K *in advanced ovarian cancer remains to be clarified. In addition, the overall survival of patients with chemo-resistance was significantly decreased with up-regulation of the genes including: *RNase K*, *FOXP1*, *LAMB2 *and *MRVI1 *(Figure 6). This might implicate these genes in chemoresistance.

## Conclusion

One hundred and fourteen DEGs were identified from 16 patients with stage III serous ovarian carcinoma using the ACP-based RT-PCR technique. Fifteen percent of the total DEGs were associated with apoptosis, the immune response, cell adhesion, and signal pathways. The genes related to apoptosis inhibitory processes tended to be up-regulated while the genes associated with the immune response tended to be down-regulated. The up- and down-regulated genes were identified in most of the patients and might be used as predictive markers in stage III serous ovarian cancer.

## Competing interests

The authors declare that they have no competing interests.

## Authors' contributions

YSK, DHB and WSA designed the study and provided the clinical background. DHB and WSA performed sample annotation and gathered follow-up of the patients. SB performed the experiments. JHD and SB carried out data analysis and JHD wrote the manuscript. All authors contributed to the manuscript and approved it.

## Pre-publication history

The pre-publication history for this paper can be accessed here:

http://www.biomedcentral.com/1471-2407/10/576/prepub

## Supplementary Material

Additional file 1 **The log_2 _expression ratios, of cancer to normal samples measured by the quantitative real-time PCR, for the 38 DEGs from 16 patients**. Click here for file
